# Supplementary material for: Assembly of a pangenome uncovers novel non-reference unique insertion sequences in cattle highlighting their genetic diversity
Source: J Anim Sci Biotechnol. 2026 Mar 9;17:47. doi: 10.1186/s40104-026-01373-3 (PMC12969903; doi:10.1186/s40104-026-01373-3)
Supplement: Supplementary file 1 — Additional file 1: Fig. S1. Abondance haplotype 1 (A) and 2 (B). Fig. S2. Aubrac haplotype 1 (A) and 2 (B). Fig. S3. Blonde d’Aquitaine haplotype 1 (A) and 2 (B). Fig. S4. Charolais haplotype 1 (A) and 2 (B). Fig. S5. Holstein-Normande haplotype 1 (A) and 2 (B). Fig. S6. Tarentaise haplotype 1 (A) and 2 (B). Fig. S7. Vosgienne haplotype 1 (A) and 2 (B). Fig. S8. Yak-Montbéliarde haplotype 1 (A) and 2 (B). [file 40104_2026_1373_MOESM1_ESM.pdf]

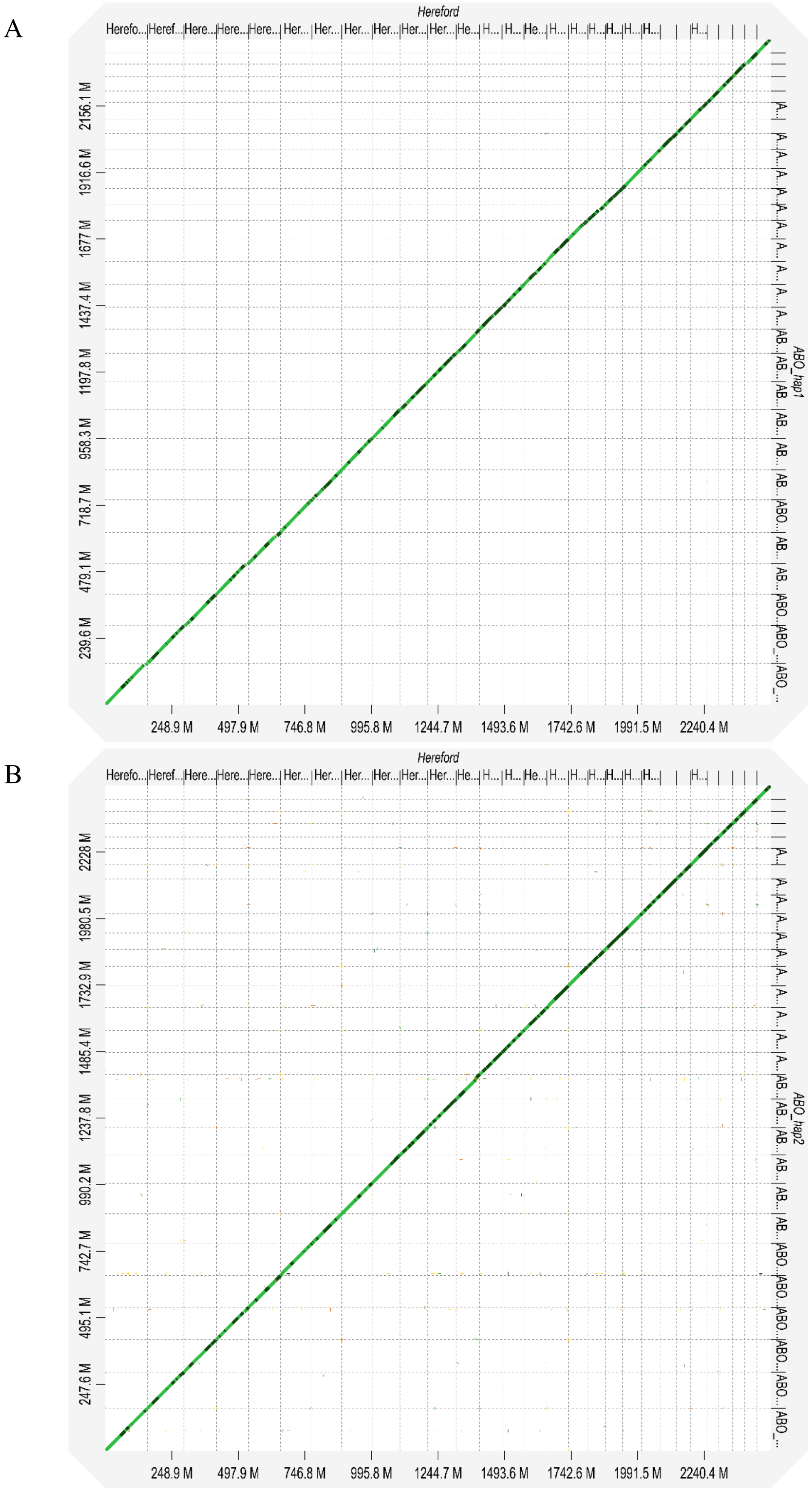

Fig. S1 D-Genies plot for chromosomal alignment concordance between ARS-UCD1.2 on x-axis and Abondance haplotype (A) 1 and (B) 2 on y-axis.

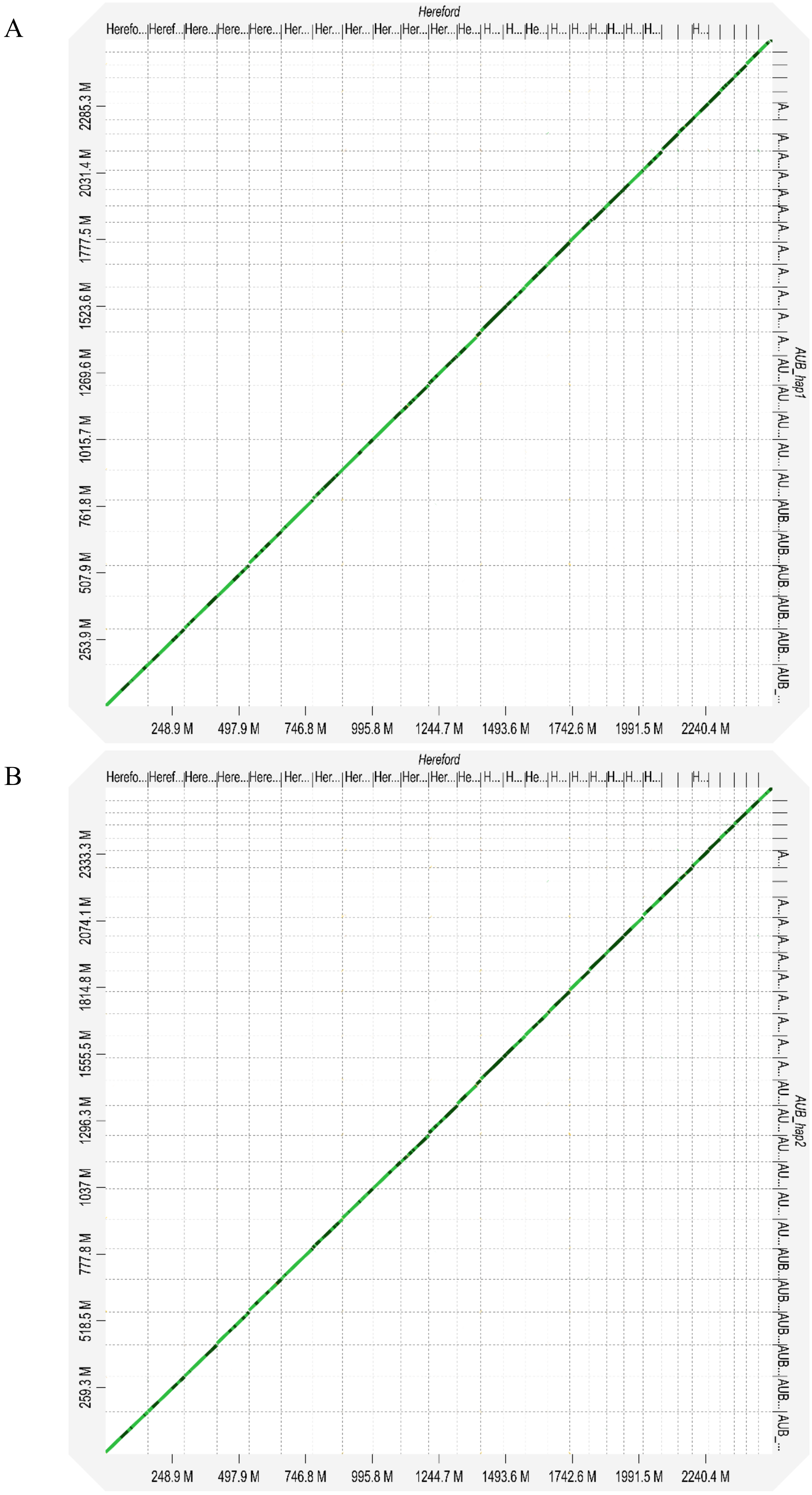

Fig. S2 D-Genies plot for chromosomal alignment concordance between ARS-UCD1.2 on x-axis and Aubrac haplotype (A) 1 and (B) 2 on y-axis.

A

B

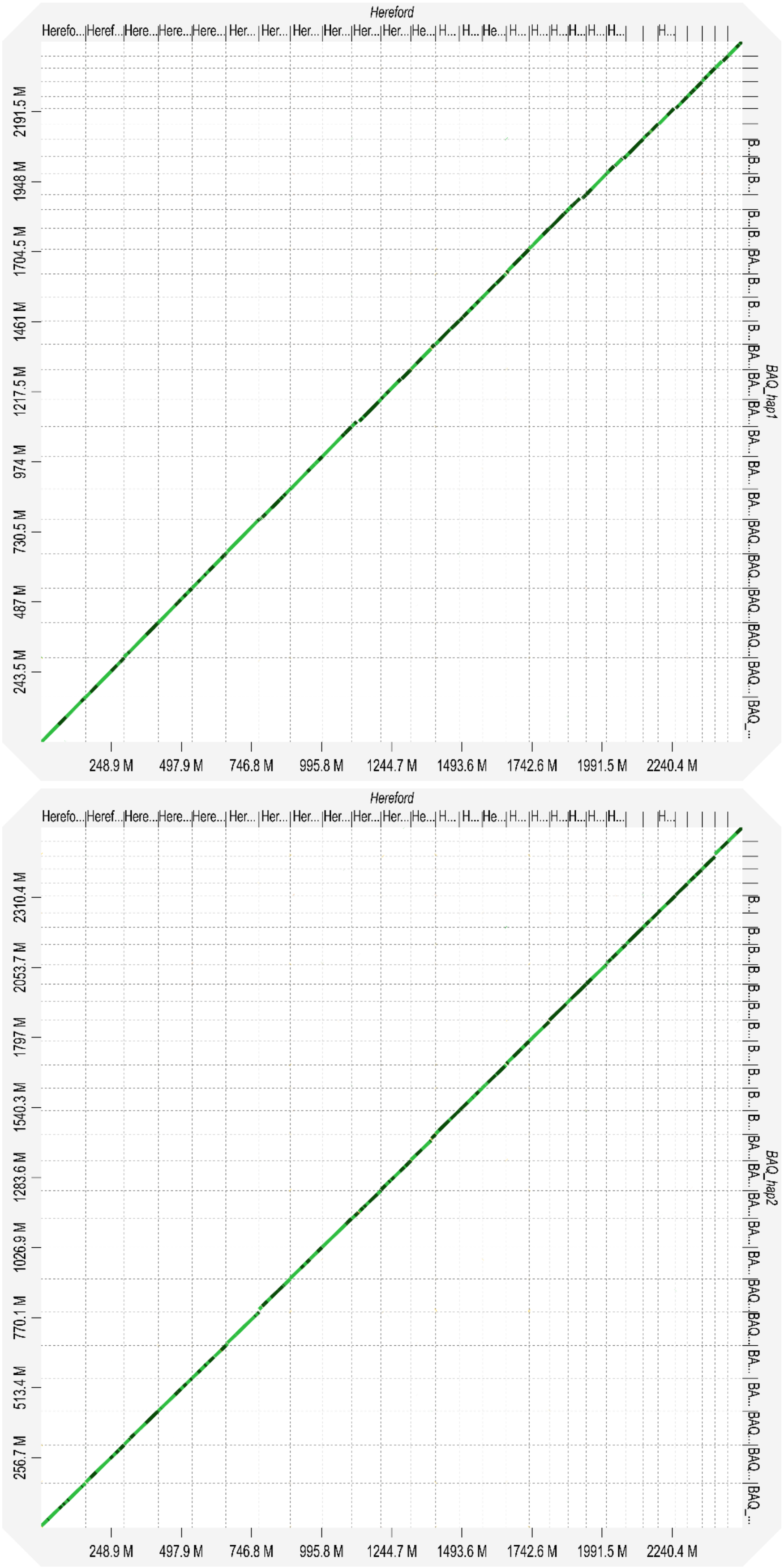

Fig. S3 D-Genies plot for chromosomal alignment concordance between ARS-UCD1.2 on x-axis and Blonde d'Aquitaine haplotype (A) 1 and (B) 2 on y-axis.

A

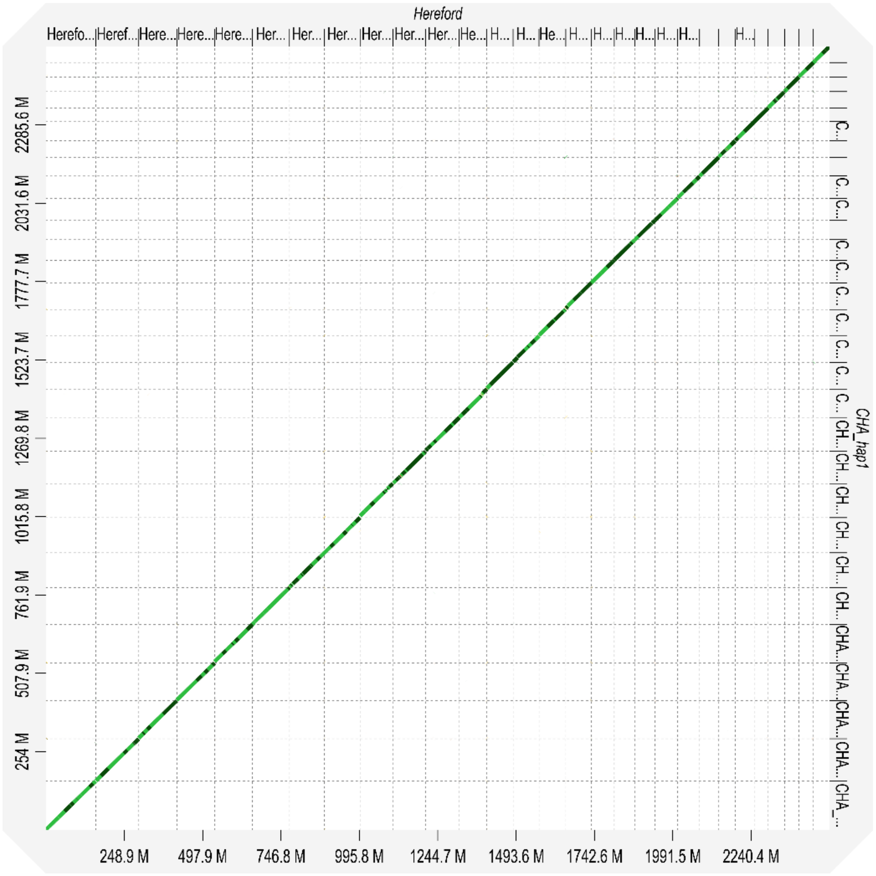

B

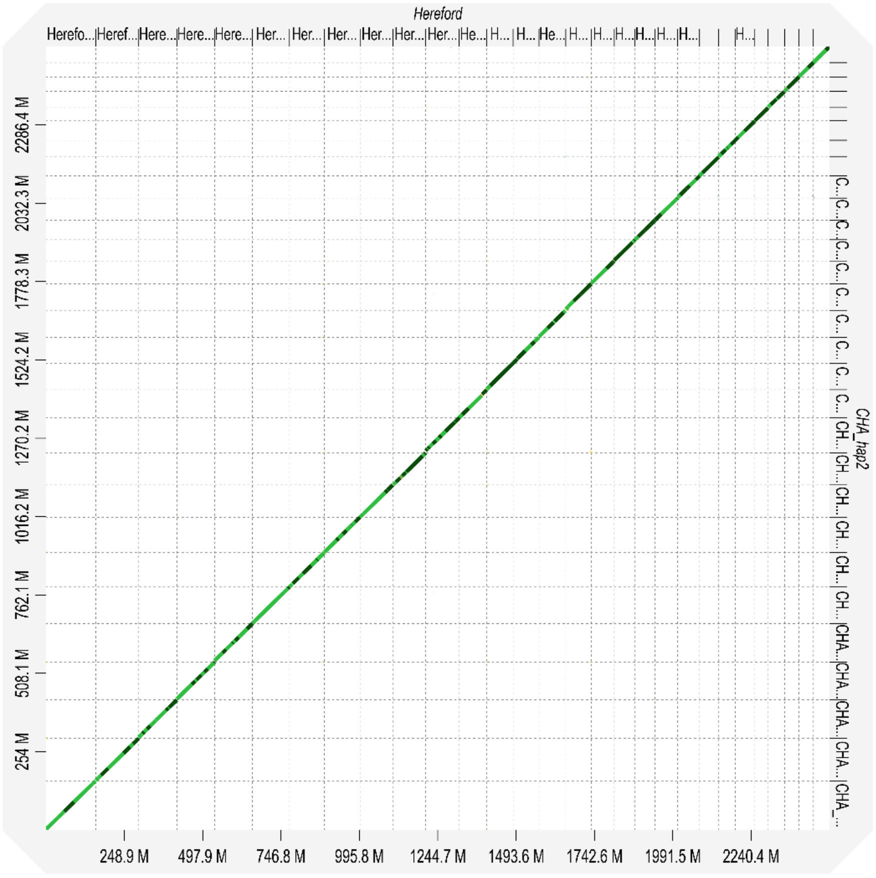

Fig. S4 D-Genies plot for chromosomal alignment concordance between ARS-UCD1.2 on x-axis and Charolais haplotype (A) 1 and (B) 2 on y-axis.

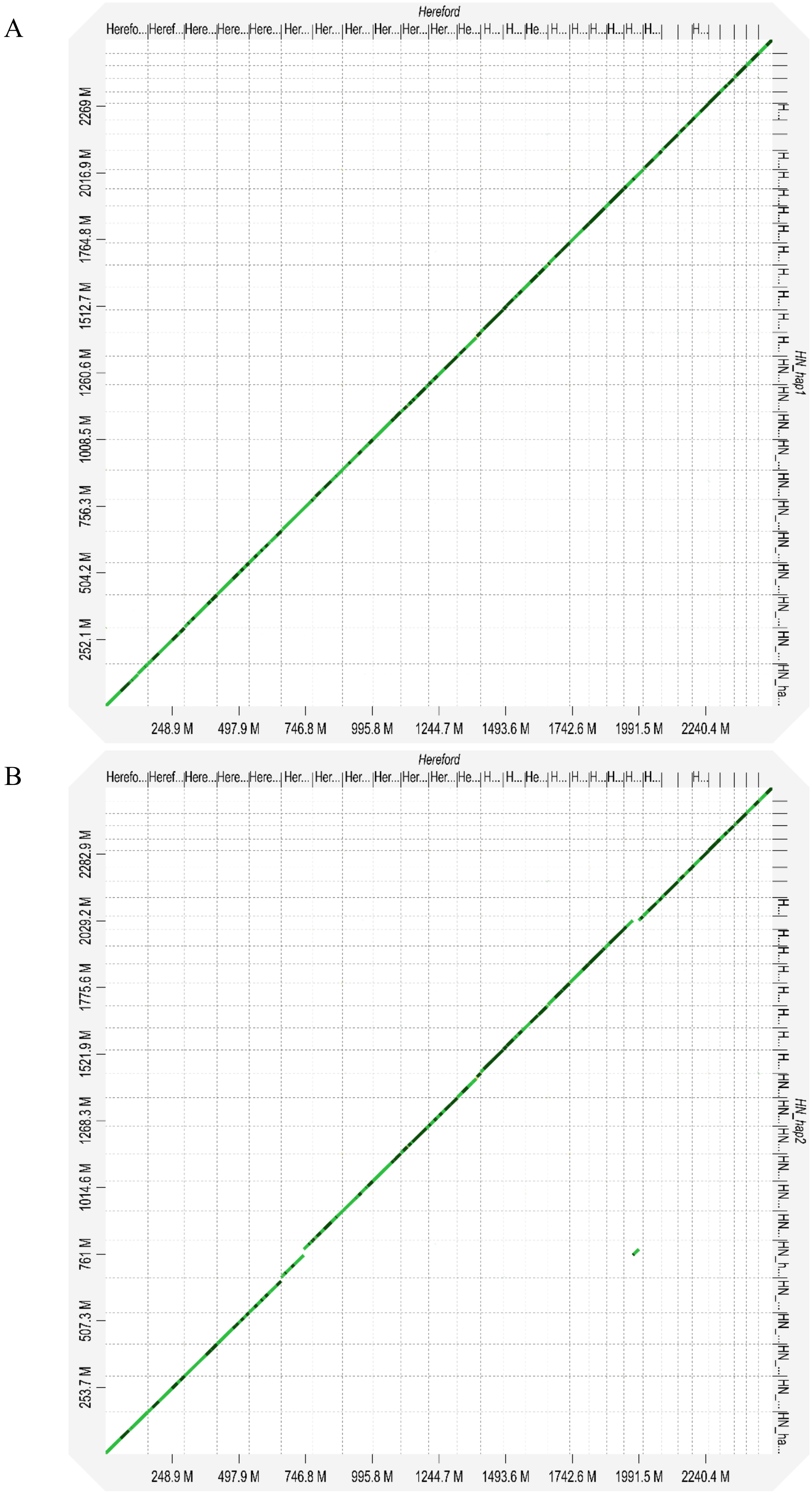

Fig. S5 D-Genies plot for chromosomal alignment concordance between ARS-UCD1.2 on x-axis and Holstein-Normande haplotype (A) 1 and (B) 2 on y-axis.

A

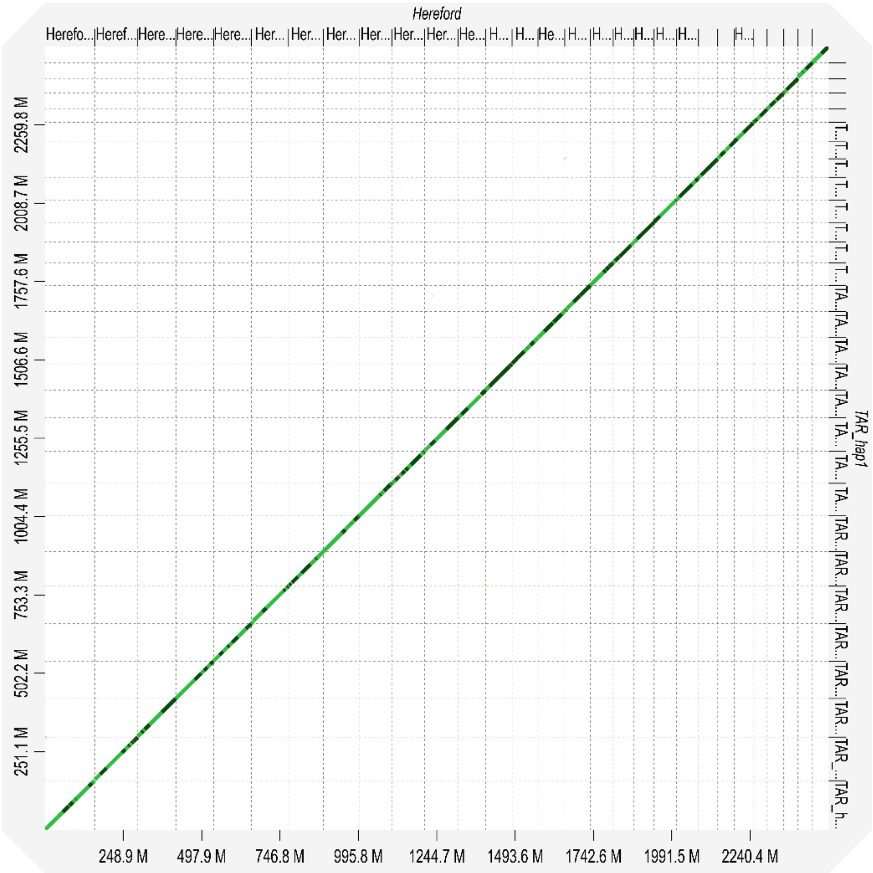

B

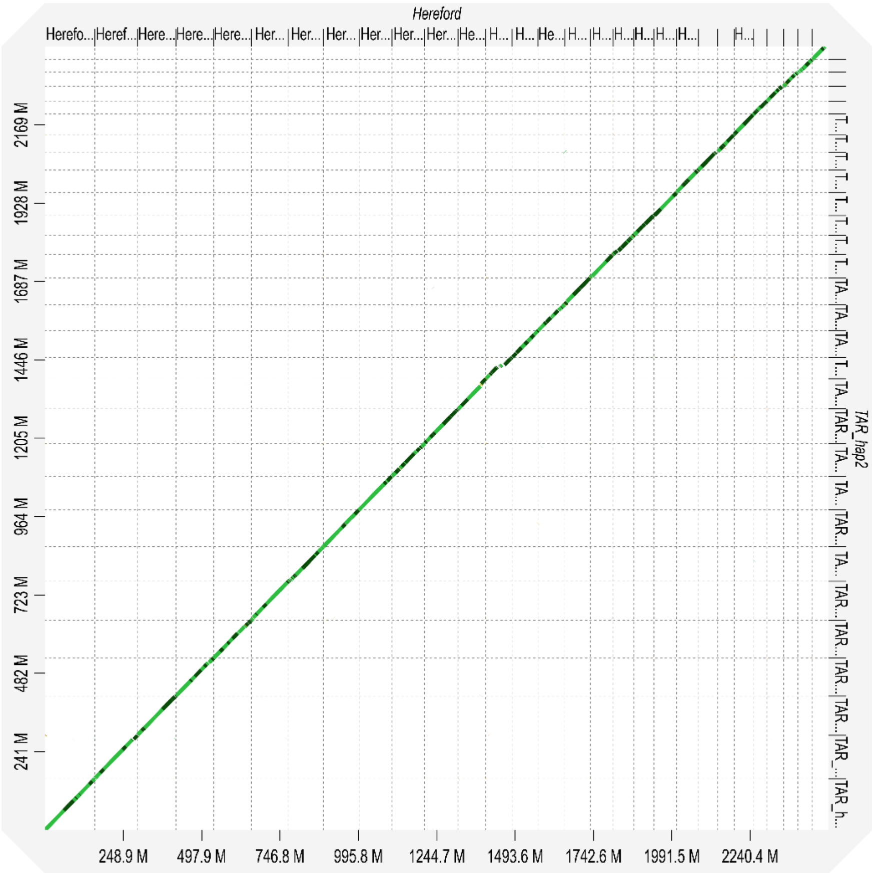

Fig. S6 D-Genies plot for chromosomal alignment concordance between ARS-UCD1.2 on x-axis and Tarentaise haplotype (A) 1 and (B) 2 on y-axis.

A

B

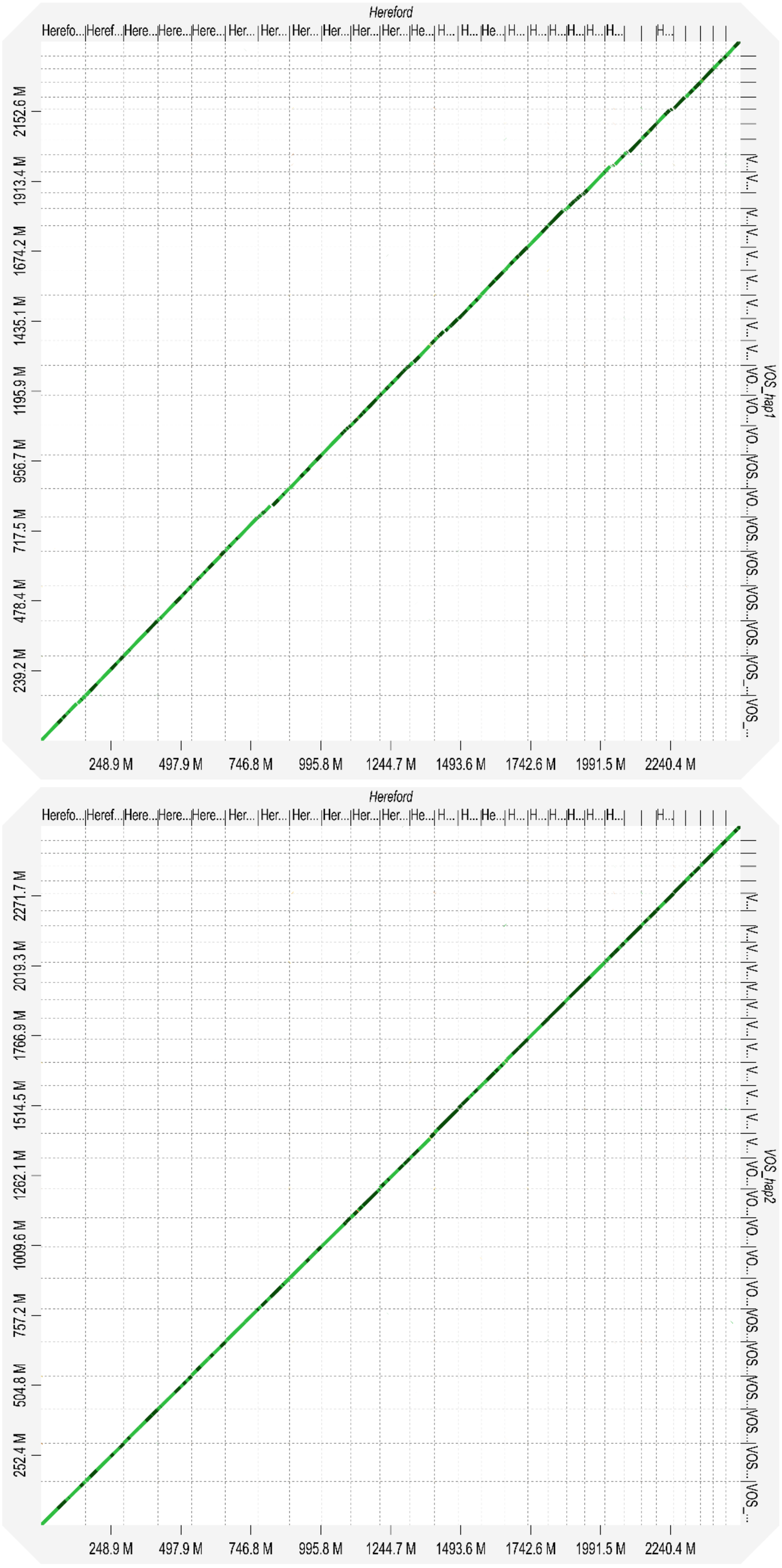

Fig. S7 D-Genies plot for chromosomal alignment concordance between ARS-UCD1.2 on x-axis and Vosgienne haplotype (A) 1 and (B) 2 on y-axis.

A

B

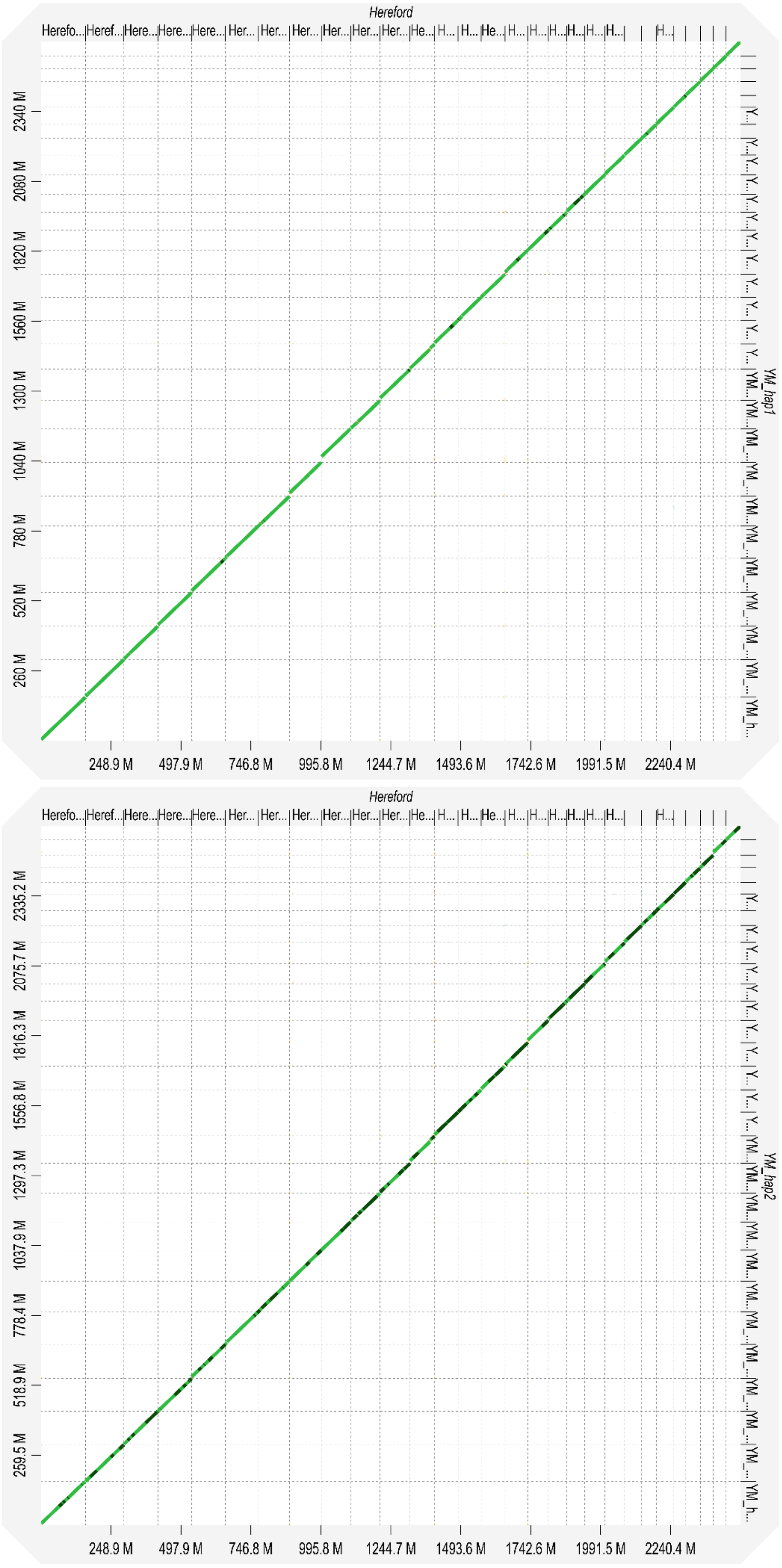

Fig. S8 D-Genies plot for chromosomal alignment concordance between ARS-UCD1.2 on x-axis and Yak-Montbéliarde haplotype (A) 1 and (B) 2 on y-axis.
