# Supplementary material for: Assembly of a pangenome uncovers novel non-reference unique insertion sequences in cattle highlighting their genetic diversity
Source: J Anim Sci Biotechnol. 2026 Mar 9;17:47. doi: 10.1186/s40104-026-01373-3 (PMC12969903; doi:10.1186/s40104-026-01373-3)
Supplement: Supplementary file 3 — Additional file 3: Fig. S9. (A) Bandage visualisation of the NRUI identified within the ARMH3 gene. (B) Gel electrophoresis of 5 samples for ARMH3 NRUI: one homozygous reference sample (40515), two heterozygous samples (40516 and 40522), and two homozygous samples for the NRUI (40517 and 40529). (C) Sanger sequencing of the ARMH3 NRUI sequence and its breakpoints. Fig. S10. (A) Bandager visualisation of the NRUI located within the EPHA5 gene. (B) Gel electrophoresis of 6 samples for EPHA5 NRUI: two homozygous reference samples (40497 and 46499), two heterozygous samples (40516 and 40522), and two homozygous samples for the NRUI (40515 and 40524). (C) Sanger sequencing of the EPHA5 NRUI sequence and its breakpoints. [file 40104_2026_1373_MOESM3_ESM.pdf]

A

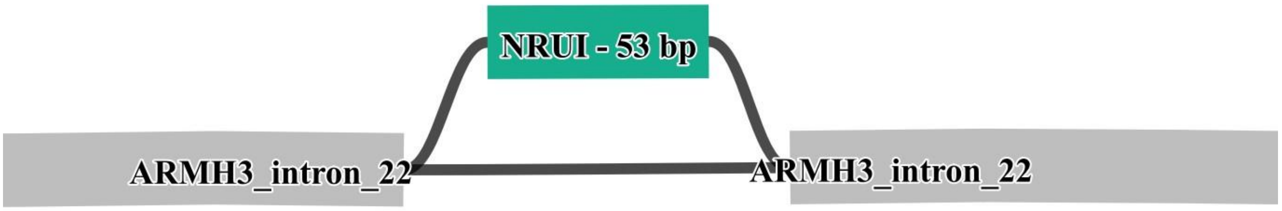

B

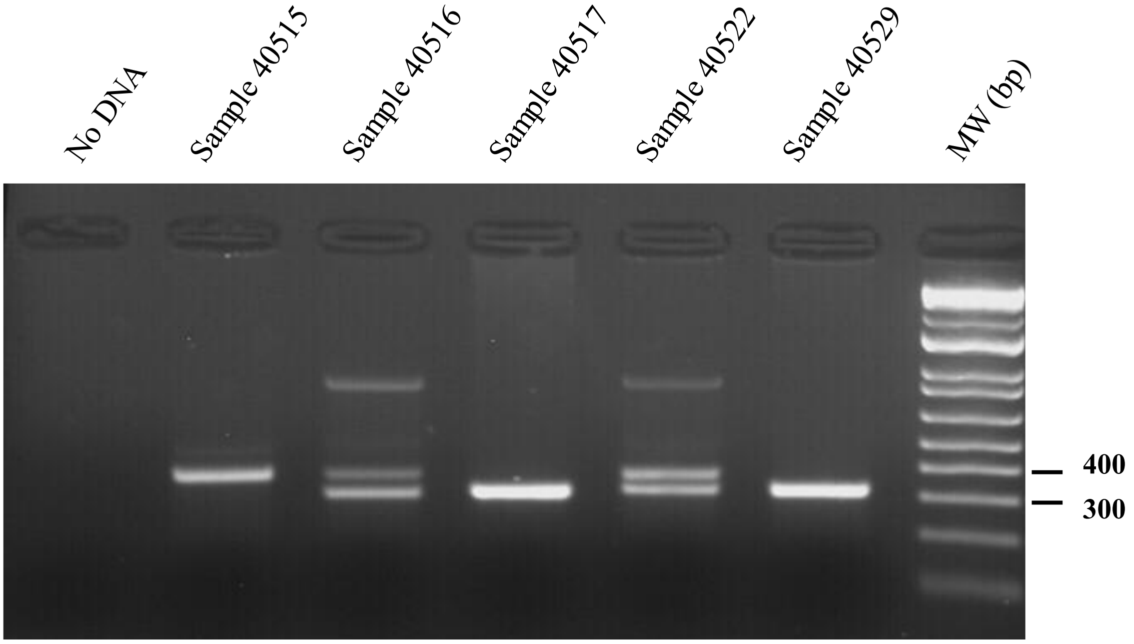

C

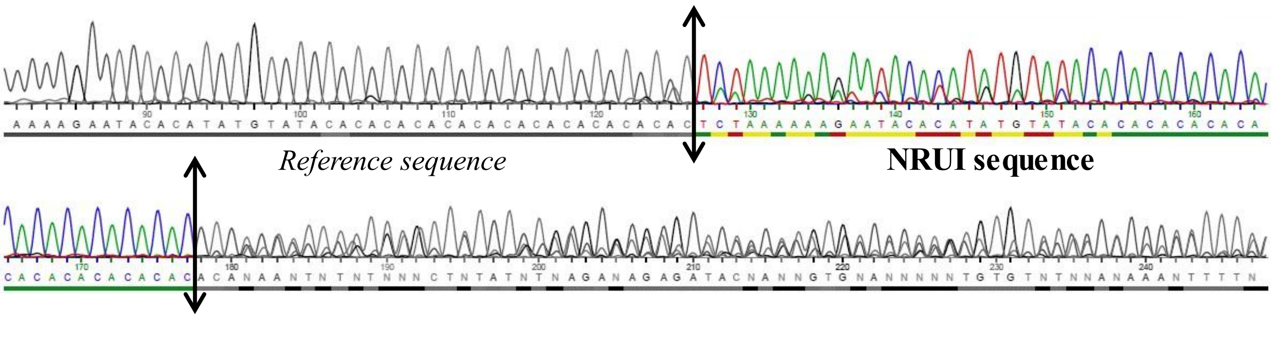

Fig. S9. (A) Bandage visualisation of the NRUI identified within the *ARMH3* gene. (B) Gel electrophoresis of 5 samples for *ARMH3* NRUI: one homozygous reference sample (40515), two heterozygous samples (40516 and 40522), and two homozygous samples for the NRUI (40517 and 40529). (C) Sanger sequencing of the *ARMH3* NRUI sequence and its breakpoints.

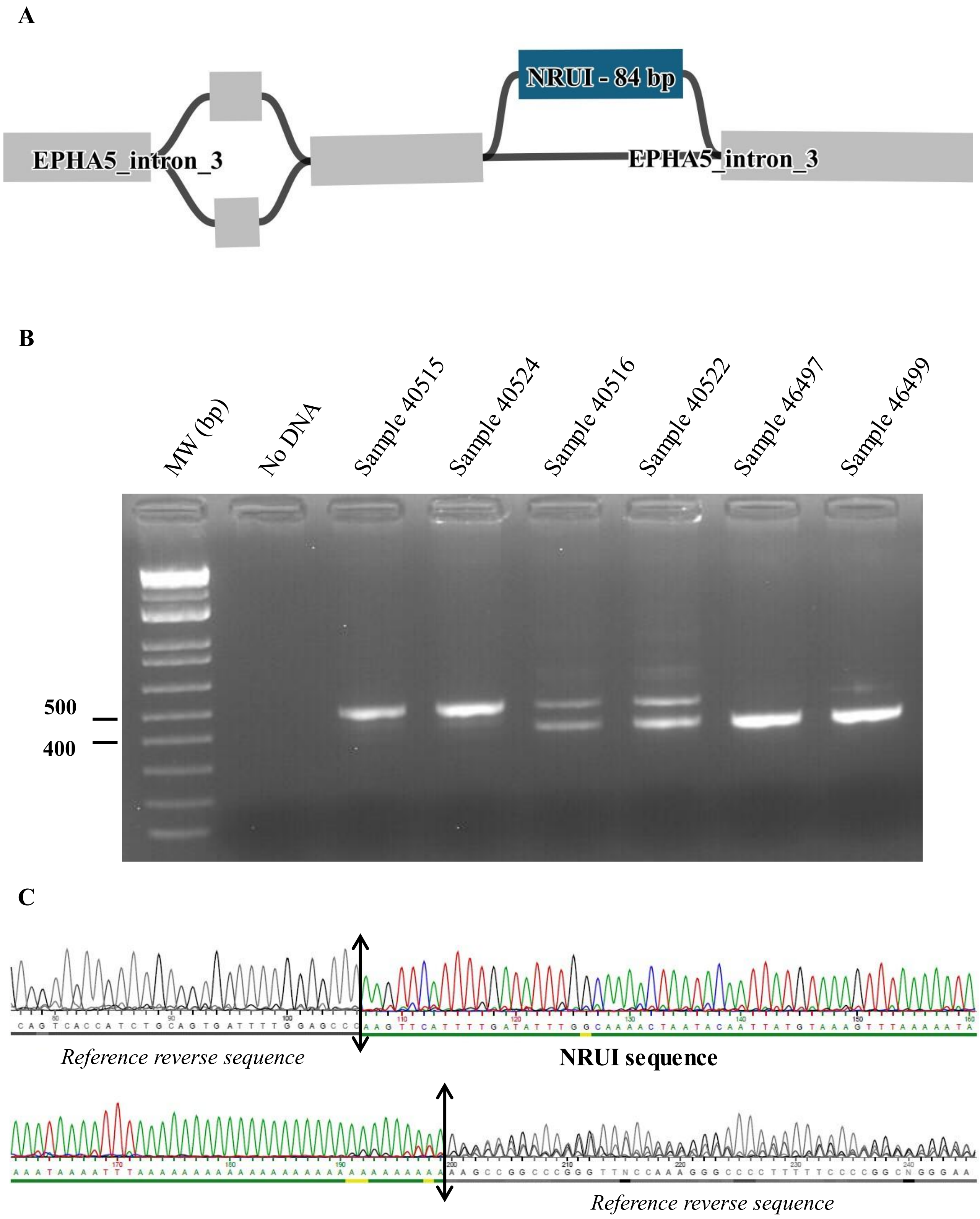

Fig. S10. (A) Bandager visualisation of the NRUI located within the *EPHA5* gene. (B) Gel electrophoresis of 6 samples for *EPHA5* NRUI: two homozygous reference samples (40497 and 46499), two heterozygous samples (40516 and 40522), and two homozygous samples for the NRUI (40515 and 40524). (C) Sanger sequencing of the *EPHA5* NRUI sequence and its breakpoints.
