# Supplementary figures and images for: Assembly of a pangenome uncovers novel non-reference unique insertion sequences in cattle highlighting their genetic diversity
Source: J Anim Sci Biotechnol. 2026 Mar 9;17:47. doi: 10.1186/s40104-026-01373-3 (PMC12969903; doi:10.1186/s40104-026-01373-3)

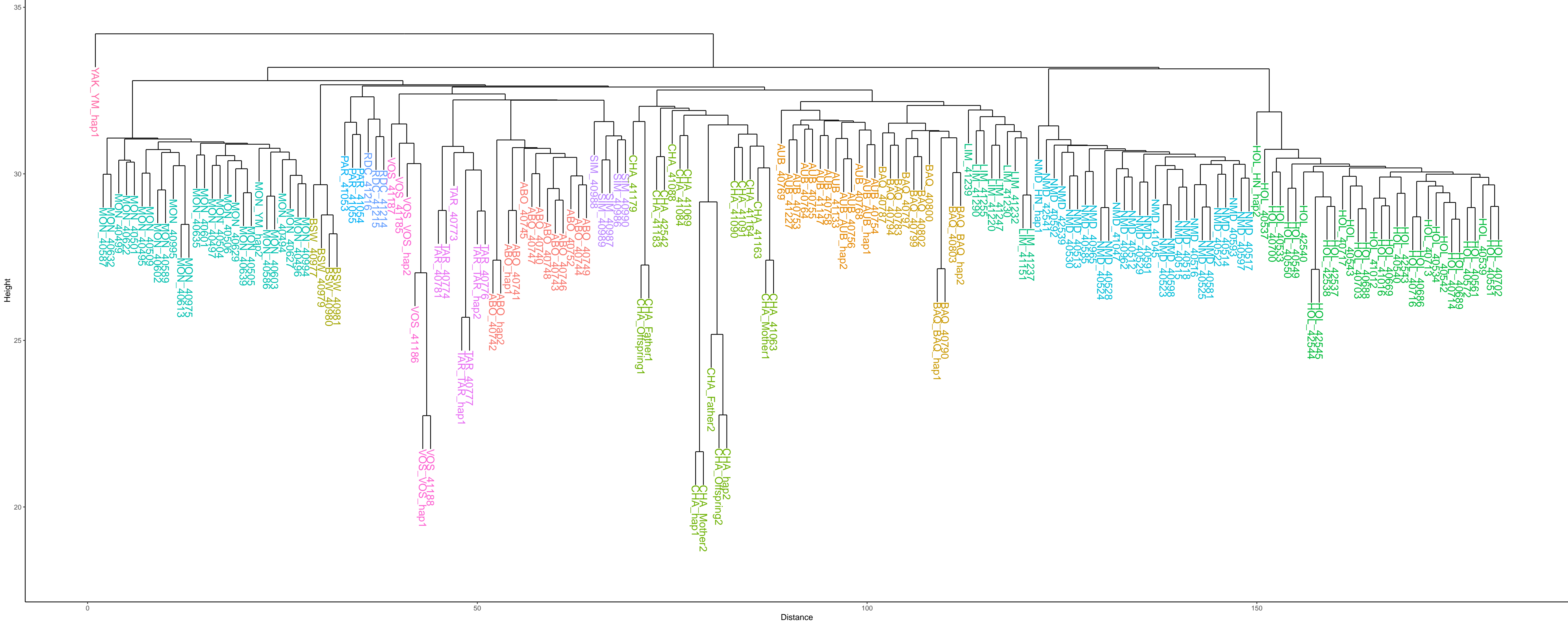

Supplement: Supplementary file 4 — Additional file 4: Hierarchical clustering of the 181 de novo assemblies based on the NRUI PAV-matrix. PAV presence/absence variation. Diagram showing the clustering of the 181 assemblies according to the 14 breeds for the 28,998 NRUIs. [file 40104_2026_1373_MOESM4_ESM.pdf]
